# Endogenous Digitalis-like Factors as a Key Molecule in the Pathophysiology of Pregnancy-Induced Hypertension and a Potential Therapeutic Target in Preeclampsia

**DOI:** 10.3390/ijms241612743

**Published:** 2023-08-13

**Authors:** Maciej W. Socha, Jakub Chmielewski, Miłosz Pietrus, Mateusz Wartęga

**Affiliations:** 1 Department of Perinatology, Gynecology and Gynecologic Oncology, Faculty of Health Sciences, Collegium Medicum in Bydgoszcz, Nicolaus Copernicus University, Łukasiewicza 1, 85-821 Bydgoszcz, Poland; 2 Department of Obstetrics and Gynecology, St. Adalbert’s Hospital in Gdańsk, Copernicus Healthcare Entity, Jana Pawła II 50, 80-462 Gdańsk, Poland; 3 Department of Gynecology and Obstetrics, Jagiellonian University Medical College, 31-501 Kraków, Poland; 4 Department of Pathophysiology, Faculty of Pharmacy, Collegium Medicum in Bydgoszcz, Nicolaus Copernicus University, M. Curie- Skłodowskiej 9, 85-094 Bydgoszcz, Poland

**Keywords:** pregnancy, pregnancy-induced hypertension, preeclampsia, molecular pathway, endogenous digitalis-like factors, cardiotonic steroids, ouabain, marinobufagenin, Na^+^/K^+^-ATPase, ADA-FAB, Digibind, DigiFab, digoxin, canrenone, Fli-1, fibrosis

## Abstract

**Preeclampsia** (PE), the most severe presentation of hypertensive disorders of pregnancy, is the major cause of morbidity and mortality linked to pregnancy, affecting both mother and fetus. Despite advances in prophylaxis and managing PE, delivery of the fetus remains the only causative treatment available. Focus on complex pathophysiology brought the potential for new treatment options, and more conservative options allowing reduction of feto-maternal complications and sequelae are being investigated. Endogenous digitalis-like factors, which have been linked to the pathogenesis of preeclampsia since the mid-1980s, have been shown to play a role in the pathogenesis of various cardiovascular diseases, including congestive heart failure and chronic renal disease. Elevated levels of EDLF have been described in pregnancy complicated by hypertensive disorders and are currently being investigated as a therapeutic target in the context of a possible breakthrough in managing preeclampsia. This review summarizes mechanisms implicating EDLFs in the pathogenesis of preeclampsia and evidence for their potential role in treating this doubly life-threatening disease.

## 1. Introduction

Hypertensive disorders of pregnancy are frequent pregnancy complications, affecting both mother and fetus and putting them at substantial risk of acute and life-long sequelae. The number of deliveries associated with hypertensive disorders has been increasing rapidly in recent years and is associated with severe obstetric morbidity [1]. According to the World Health Organization (WHO), hypertension is the second most common direct cause of maternal death during pregnancy worldwide (14%) [2].

ACOG distinguishes four manifestations of hypertensive disorders of pregnancy, including chronic hypertension, gestational hypertension, preeclampsia-eclampsia, and chronic hypertension with superimposed preeclampsia [3]. According to ISSHP recommendations, preeclampsia is defined as new-onset hypertension at or after 20 weeks of gestation with at least one of the following: proteinuria, evidence of another maternal organ, or uteroplacental dysfunction. As PE can deteriorate rapidly, the former distinction between mild and severe preeclampsia is no longer used. ISSHP recommends terms PE with or without severe features, such as systolic blood pressure >160 mmHg or diastolic blood pressure >110 mmHg, or manifestations of multi-organ failure such as acute kidney injury, liver involvement, neurological, and hematological complications [4].

Preeclampsia, the most severe hypertensive disorder of pregnancy, affects 5–7% of all pregnancies and is responsible for over 70,000 maternal and 500,000 fetal deaths worldwide annually. It is the leading cause of maternal death, severe maternal morbidity, maternal intensive care admissions, cesarean sections, and prematurity [5]. In addition to acute complications, preeclampsia is associated with substantial long-term risk for cardiovascular disease (CVD) and cerebrovascular disease [6] and appears to be considered an independent cardiovascular risk factor both in mothers [7,8,9] and infants [10,11].

The management of PE consists of symptomatic treatment with antihypertensives and supportive care focused on reducing neonatal respiratory distress syndrome, namely antenatal steroids before 34 weeks of gestational age and magnesium sulfate as eclampsia prophylaxis as well as neuroprotection [4,12]. In most severe cases, expedited delivery remains the only treatment available.

Recent advances in managing preeclampsia were made in clinical practice with effective screening and prophylaxis as well as biomarkers of angiogenic imbalance, allowing for an early and more accurate diagnosis. Universal first-trimester screening for preterm PE has been implemented in clinical practice [13]. The combined test includes maternal risk factors, mean arterial pressure (MAP), serum placental growth factor (PLGF), and uterine artery pulsatility index (UtA Pi) [14]. The risk calculator is available on the Fetal Medicine Foundation website [15]. Following first-trimester screening for preterm PE, women identified as high risk (>1 in 100) should receive aspirin prophylaxis beginning before 16 weeks of gestation at a dose of 150 mg to be taken every night until 36 weeks of gestation. In the ASPRE trial, the intervention reduced the incidence of preterm PE by 62% [14]. Calcium supplementation in women with low calcium intake is recommended by ISSHP and FIGO [4,13] as the intervention may reduce the risk of preeclampsia [16]. The development of biochemical markers allows for early and more accurate diagnosis even before the onset of full clinical manifestation. The sFlt-1/PlGF ratio is used as a tool for short-term prediction and as an aid for diagnosis in high-risk women or among women with a clinical suspicion of preeclampsia [17].

Despite advances in screening, prophylaxis, and early diagnosis, the need for causative treatment remains unfulfilled. This brings to light the complex pathogenesis of preeclampsia. The following review focuses on endogenous digitalis-like substances, a group of steroids that have digitalis-like activity, act as regulators of Na^+^/K^+^-ATPase, and induce vasoconstrictive and natriuretic effects [18,19], also referred to as cardiotonic steroids or endogenous CS, and their contribution to the development of preeclampsia, as well as a potential target for novel therapeutic agents. Recent studies provide substantial evidence of EDLF implication in preeclampsia-related vascular fibrosis, and novel therapeutic agents can potentially alleviate not only acute feto-maternal complications but also prevent lifelong sequelae and increased cardiovascular risk associated with preeclampsia [19].

## 2. Pathogenesis of Preeclampsia

The development of preeclampsia progresses according to a two-stage model. The classical two-stage model introduced in 1991 implies that the primary disease of the placenta secondarily affects maternal organs. Stage 1 is characterized by an absence of clinical symptoms resulting in overt maternal syndrome in Stage 2 [20]. Although this concept has evolved over the years, the placenta remains central to the pathogenesis of preeclampsia.

As the original two-stage model of preeclampsia was mainly applicable to early-onset preeclampsia, the revised two-stage model of preeclampsia aims to unify pathogenesis and explain different clinical manifestations of PE, namely early-onset and late-onset PE, as well as rare forms of postpartum preeclampsia and atypical preeclampsia with overt symptoms developing before 20 weeks of gestational age. In early-onset preeclampsia, the cytotrophoblast fails to adapt to the invasive endothelial subtype, which leads to impaired trophoblast invasion, shallow placentation, and incomplete spiral arteries remodeling, resulting in more pulsatile and higher pressure flow, which is predicted to damage the chorionic villi mechanically as well as through generating ischemia-reperfusion injury and placental oxidative stress [21]. Maladapted arteries are prone to atherosis, further restricting placental flow. Defective placentation may result from factors affecting both the trophoblast and the decidua. Poor uterine decidualization, atherosclerotic changes in maternal radial arteries, and decidual vasculopathy (DV) are also often observed in PE [5]. Late-onset preeclampsia results from uteroplacental malperfusion, without evidence of poor placentation, when the placenta outgrows uterine capacity. Both mechanisms lead to syncytiotrophoblast hypoxia and stress and can be affected by maternal factors predisposing to increased inflammation [22]. The ischemic placenta subsequently releases antiangiogenic and proinflammatory factors into the maternal circulation, including cytokines, reactive oxygen species (ROS), and the angiotensin II type 1 receptor autoantibody (AT1-AA), among many others [23]. These substances may cause widespread endothelial activation, up-regulation of the endothelin system, and vasoconstriction. In turn, these changes affect the function of multiple organ systems, including the kidneys, brain, liver, and heart [23]. A more detailed discussion regarding the mechanisms mentioned above implicated in the pathogenesis of preeclampsia is beyond the scope of the present paper.

Although the primary cause of the cascade of events during the development of preeclampsia remains elusive, alterations in immunological response play a major role in the pathogenesis of preeclampsia, with the most critical mechanisms being an aberrant proinflammatory shift towards Th1 response, decreased level of uNK, complement dysregulation. Genetic and environmental factors are also linked to the development of preeclampsia [24]. Uterine NK cells express killer immunoglobulin-like receptors (KIR), which bind to HLA-C molecules on EVT. As genes encoding maternal KIR and fetal HLA-C are highly polymorphic, extravillous trophoblast (EVT) interactions with uNK cells are an important determinant of the risk of preeclampsia. Other genetic influences affect the stress response in the placenta and the maternal susceptibility to systemic syndrome [25]. Major risk factors for PE include chronic hypertension, pregestational diabetes mellitus, obesity, antiphospholipid syndrome, and a history of PE in a previous pregnancy [26].

## 3. Na^+^/K^+^-ATPase- “Sodium Pump”

The Na^+^-plus-K^+^-dependent adenosine triphosphatase, commonly referred to as the “sodium pump,” is ubiquitous and highly conserved in all eucaryotes and is an active transport system of sodium and potassium. This transmembrane protein is responsible for maintaining the low Na^+^/K^+^ ratio by active transport across the plasma membrane using energy derived from the hydrolysis of ATP. The Na^+^/K^+^-ATPase (NKA) controls multiple cellular functions, with maintaining the electrical transmembrane potential being the most obvious one, as well as driving secondary transporters such as Na^+^/Ca^2+^-exchanger [27].

The Na^+^/K^+^-ATPase is composed of two subunits: catalytic subunits and glycosylated glycoprotein subunits, with molecular masses of 110 kDa and 31.5 kDa, respectively. The α subunit has 10 transmembrane segments with extracellular binding sites for Na^+^ and CS and intracellular binding sites for K+ and ATP. The β subunit has a regulatory function, with the glycosylation site controlling the enzyme’s activity. Complexes form functional dimeric units [27,28]. NKA interacts closely with regulatory transmembrane peptides called FXYD proteins [29].

The catalytic cycle of the Na^+^/K^+^-ATPase results in “pumping” sodium ions outside the cell while moving potassium ions inside the cell, both against concentration gradients. According to the Albers–Post model, the α subunit in the presence of sodium is phosphorylated by ATP and occludes three cytosolic Na^+^ ions. This forms the high-energy E1P state, which then undergoes a conformational change to the low-energy E2P form and releases sodium ions outside the cell. In the presence of potassium ions, the E2P form is dephosphorylated. Dephosphorylation is followed by the occlusion of two extracellular K^+^ ions. Uptake of potassium leads to the transition from E2 to E1 form, which is accelerated by ATP and followed by the release of two potassium ions inside the cell. The E1 form is bound with ATP, and the enzyme undergoes the cycle again [27].

Mammals express four α (α1, α2, α3, α4), three β (β1, β2, β3) and seven FXYD subunit isoforms. The different isoforms have various kinetic properties and affinities and are expressed in a tissue-specific fashion. The α1β1 complex is expressed most widely [30]. The distribution of α1 isoform is ubiquitous; it is also the primary isoenzyme in the vascular wall and kidney [27,31]. α2 isoform is predominantly expressed in the heart, vascular smooth muscle, and skeletal muscle, as well as in the brain, specifically in astrocyte and glia cells; α3 is expressed mainly in excitable tissues, including neurons and the conductive system of the heart; and α4 subunit is sperm-specific. β1 is omnipresent, and β2 and β3 are expressed in the brain and erythrocytes, among many others [27].

Endogenous Na^+^/K^+^-ATPase inhibitors, cardiotonic steroids (CS), presented in detail in the following section, have a specific binding site on the extracellular loops of the NKA α subunit (TM1-TM2, TM5-TM6, and TM7-TM8); also some amino acids from the transmembrane regions interact with ouabain. The sensitivity of the sodium pump to cardiotonic steroids is controlled by multiple mechanisms in addition to the tissue specificity of the α and β isoforms distribution. Protein kinases phosphorylate NKA in a tissue- and isoform-specific manner [27]. At low concentration levels, CS interacts with NKA in a ligand-receptor manner rather than as its inhibitor, initiating several intracellular signaling pathways [32].

## 4. Endogenous Digitalis-Like Factors (EDLF)

### 4.1. General Information

Endogenous digitalis-like factors (EDLF) are a group of steroids that have digitalis-like activity and act as regulators of Na^+^/K^+^-ATPase (the sodium pump) [19]. Cardiotonic steroids bind to the α subunit of the NKA and induce vasoconstrictive and natriuretic effects [18]. Endogenous CS levels are known to increase in response to volume expansion states accompanying chronic diseases such as hypertension, heart failure, and chronic kidney disease and are implicated in mediating both adaptive and maladaptive responses to volume overload [33,34,35]. Chronic elevation of these compounds may contribute to disease progression, potentially via profibrotic and proinflammatory effects [36].

Two classes of compounds recognized as EDLF have been identified: 1-analogs to plant-derived cardenolides, which include ouabain, digoxin, digitoxin, and 2-bufadienolides, such as those extracted from the venom of Bufo marinus toad which includes marinobufagenin, as well as telcinobufagenin, bufalin, and proscillardin [36]. These two classes differ structurally in the number of atoms in the lactone ring located at the C17 position on the steroid nucleus: a 5-member unsaturated butyrolactone ring in cardenolides and a 6-member unsaturated pyrone ring in bufadienolides [37,38]. Although the biosynthesis of endogenous CS remains to be fully explained, different pathways for endogenous ouabain (EO) and marinobufagenin (MBG) have been described. The “classic” steroid hormone synthesis pathway is implicated in the biosynthesis of cardenolides (EO), while bufadienolides (MBG) are derived from the transformation of bile acid. 

The side chain cleavage of cholesterol via P450scc (CYP11A1) is essential for the synthesis of EO. Although synthesis of pregnenolone followed by conversion to progesterone by δ5-3β-hydroxysteroid dehydrogenase has been identified as the next step in EO biosynthesis, enzymes inverting configuration at carbons 5 and 14 to form a cis-trans-cis configuration are yet to be identified [39,40]. There is evidence for the neuroregulatory role and neuroendocrine source of endogenous ouabain [41,42], and the adrenal cortex and hypothalamus are considered to be important sites of EO production [43].

The biosynthesis of bufadienolides does not involve the pathway implicated in the synthesis of “classic” steroid hormones and does not require cholesterol side chain cleavage. The transformation of bile acids using the P450 family enzyme CYP27A1 is implicated in the biosynthesis of MBG. MBG has been found to be produced in the adrenal cortex and placenta [44].

Endogenous digitalis-like factors exhibit differential affinity for different α-subunits of NKA. Thus, α1 NKA, the isoform predominant in vascular smooth muscle and kidney, is inhibited by physiologically relevant concentrations of MBG, while α2 and α3 NKA are more sensitive to EO [31,45,46,47]. 

EDLF is bound to plasma proteins and exists in three states in serum: tightly bound, weakly bound, and unbound (free). In normal subjects, more than 90% of the total EDLF in serum is tightly but reversibly bound to serum proteins, limiting its ability to react with antibodies. As a result, a significant fraction of EDLF is not readily detectable by direct measurement with conventional immunoassays. However, in pregnant women and some diseases, there seems to be a redistribution toward weakly bound and unbound components [48]. 

Total EDLF concentrations in healthy human subjects, as determined using antibodies, range between 0.5 to 2 nM, which is comparable to levels of other steroid hormones in human circulation [37]. Anti-EO and anti-MBG antibodies are highly specific and do not cross-react with marinobufagenin and ouabain, respectively [37]. However, commercial preparations of Fab fragments of anti-digoxin antibodies (ADA-FAB) marketed for the treatment of digoxin toxicity, Digibind@ (GlaxoSmithKline, Brentford, UK) and DigiFab@ (BTG International, Ltd, London, UK), have been found to cross-react with both EO and MBG [49] (Table 1). However, this cross-reactivity appears relatively low: 0.39% for ouabain and 0.11% for marinobufagenin, as determined for Digibind@ [47]. This characteristic of ADA-FAB was first used in preeclamptic patients by Goodlin in 1988 [50]. It was followed by numerous in vitro studies, studies on animal models of preeclampsia, and clinical trials that investigated the potential therapeutic effects of immunoneutralization of endogenous digitalis-like factors.

### 4.2. Cardiotonic Steroids in Normal Pregnancy

As normal pregnancy is associated with fluid and sodium retention [51], the attention around the possible role of EDLF in pregnancy arose after their discovery [52]. In 1984, two research teams found an endogenous factor that cross-reacted with digoxin antibodies in the serum of pregnant women, and increased levels of this factor were associated with hypertensive disorders of pregnancy [53,54]. Subsequently, an ouabain-like factor was reported in the amniotic fluid of third-trimester women. Levels of this compound were higher in hypertensive pregnant women when compared to the normotensive group and correlated with diastolic blood pressure at the time of amniocentesis [55]. These findings stimulated further studies that aimed to establish the role of endogenous digitalis-like factors both in normal and hypertensive pregnancy. 

Notably, most older studies were performed using immunoassay techniques with polyclonal and monoclonal digoxin antibodies that either do not cross-react at all with EDLF or cross-react to varying degrees. Moreover, most studies performed assays on unheated samples, thus measuring only the unbound and weakly bound forms of EDLF. This resulted in considerable variations in the concentration of EDLF reported by different laboratories [19]. That said, older studies will be summarized briefly, focusing more on recent works.

EDLF is detectable in normal pregnancy since the first trimester, with serum levels increasing progressively with gestational age [56,57,58,59,60,61]. As mentioned above, higher levels of EDLF during pregnancy are ascribable to increased amounts of free and weekly bound fractions [48], and these EDLF fractions measured in pregnant females are up to six times higher than in controls. This difference was reduced in samples measuring total EDLF level, i.e., after heating the specimen [19]. Both EO [47,49,62,63,64,65] and MBG [47,49,62,64,66,67] were detected in normal human pregnancy using immunoassays; MBG was also identified in the plasma of both pregnant and non-pregnant women utilizing LC-MS assay [68]. MBG levels in healthy pregnant females were twice higher than in normal non-pregnant adults, while EO levels were not altered [62]. Furthermore, MBG levels were 30–90% higher than EO levels in normal pregnant females [47,49,62,64].

EDLF are cleared rapidly from the maternal circulation following delivery to undetectable levels after 24 h [53]. The rapid disappearance of EDLF from maternal blood after birth suggests its fetoplacental origin [19]. Placenta is a proposed important site for producing cardiotonic steroids during pregnancy [69]. Several studies support the placental origin of EDLF, where digitalis-like immunoreactivity was identified in placental tissue [18,69,70,71,72,73]. Moreover, MBG immunoreactivity has been detected in normal human placenta [74], and MBG was isolated using high-performance liquid chromatography (HPLC) [18]. Furthermore, a synthetic pathway for MBG has been demonstrated in human placental cell culture [44].

Neonatal EDLF levels, in contrast to maternal, are up to ten times higher, persist longer after delivery, and are detectable in virtually 100% of cases. During the delivery process, the burst of EDLFs in neonatal circulation is observed and may be linked to the stress of the delivery [19]. This hypothesis is supported by a study showing lower newborn EDLF levels after cesarean section compared with vaginal delivery [75] and the observation that fetal EDLF levels before delivery correlate with catecholamine levels [76]. A comparison of umbilical artery and veins levels at birth showed no difference or slightly higher levels in the umbilical vein. The lack of AV difference suggests the surge in production at birth is likely fetal rather than placental [19].

While the precise identity of EDLFs remains to be confirmed, ouabain and marinobufagenin (MBG) have been proposed as likely EDLFs elevated in pregnancy and preeclampsia [77]. Their role in physiological pregnancy is yet to be elucidated. Normal pregnancy is characterized by renal sodium retention and plasma expansion in the presence of decreased blood pressure [78], and EDLF may play a role in adaptation to these changes. While in normal pregnancy, MGB levels are not high enough to exhibit relevant vasoconstrictive effects [79], MBG might balance the influence of vasodilator factors associated with pregnancy [19]. Some studies indicate that EO contributes to maintaining normal blood pressure during pregnancy [80] or is a growth factor necessary for fetal growth and development [65].

### 4.3. Endogenous Digitalis-like Factors in Preeclampsia

#### 4.3.1. Endogenous Digitalis-like Immunoreactivity in Preeclampsia

As an endogenous substance that cross-reacts with digoxin was demonstrated in pregnancy, the elevated levels of this digoxin-like immunoreactivity were found in subjects with preeclampsia, and the idea that these compound plays a role in the pathophysiology of preeclampsia arose [54]. Subsequently, similar observations were made by other authors. Studies confirmed elevated levels of digitalis-like immunoreactivity and elevated Na^+^/K^+^-ATPase inhibitory activity in PE [54]. In a recent study, serum EDLF levels doubled in patients diagnosed with PE compared to healthy pregnancies [81].

Markedly elevated serum levels of EDLF in patients diagnosed with preeclampsia were reported, with even higher levels in women who developed eclampsia [54,60,82,83,84,85]. EDLF levels correlate with the severity of PE [47,62]. Elevated blood levels of EDLF substantially decreased sodium pump activity; severe preeclampsia was associated with a 43% decrease in NKA activity compared to normotensive pregnancy [86]. Cord blood levels of EDLF were also significantly higher in PE than in normal pregnancy, reaching levels two times higher than in maternal blood [60,82,83,87].

As mentioned above, the placenta is the site of the synthesis of cardiotonic steroids during pregnancy. Explants cultures of placental tissue obtained at delivery produced higher levels of EDLF when acquired from a pregnancy complicated with preeclampsia than from uncomplicated pregnancy [69]. The development of PE is associated with the heightened sensitivity of the placental sodium pump to digitalis [88]. Placental homogenates obtained from preeclamptic women exhibited higher sodium pump inhibition than normotensive controls, which was reversed by ADA-FAB (Digibind@) [73].

Summarizing the presented data, we conclude that elevated level of EDLF is associated with the occurrence of preeclampsia and results in substantial inhibition of NKA activity both in serum and placenta.

#### 4.3.2. MBG as the Main Cardiotonic Steroid Implicated in the Pathogenesis of PE

After the establishment of specific assays for both groups of CS—cardenolides and bufadienolides— in most studies, significantly elevated serum levels of MBG but not EO were confirmed in preeclamptic subjects [27]. In severe preeclampsia (average blood pressure 160/104 mmHg), both ouabain and MGB plasma levels were increased as high as fourfold and eightfold, respectively [62]. However, in studies where patients with mild preeclampsia were enrolled, elevation on EO was below statistical significance [47,49]. Patients with preeclampsia in mean gestational age 37 weeks exhibited plasma concentration of MBG increased twofold [47,67] and threefold [64]. MBG levels in blood and urine samples obtained from preeclamptic subjects were significantly higher than normotensive controls; MBG elevation in both specimens was observed at various gestational age periods and in the mean level of this compound. As results in blood and urine samples were comparable, the researchers concluded that MBG levels in urine samples might be a novel approach in screening tests for preeclampsia [66].

Development of PE is associated with an even more significant increase in MBG placental levels. In a study where patients with PE in mean 39 weeks of gestational age were enrolled, placental MBG was markedly elevated, reaching ten times higher values than normotensive control [74,89]. Other studies reported two- [67], three- [64], and four-fold [18] elevation of MBG in preeclamptic placentae when compared to normal. In two of these studies, EO in preeclamptic placentae exhibited an increase in borderline statistical significance, suggesting that bufadienolides rather than cardenolides represent pathophysiologically-relevant cardiotonic steroids in preeclampsia [18,64].

Increased MBG plasma level is associated with significant inhibition of sodium pump activity [47,49,64,89]. Commercially available ADA-FAB (Digibind@ and DigiFab@) and anti-MBG antibodies restored NKA activity [49,64]. Digibind was less effective in restoring NKA activity than anti-MBG [47,64]. At the same time, pretreatment of the erythrocytes with anti-ouabain antibodies did not reverse the inhibition of the enzyme [47]. Given the above observations, MBG, rather than EO, appears to be a primary target for immunoneutralization in PE.

### 4.4. EDLF and Pathophysiology of Preeclampsia

Numerous potential mechanisms of EDLF apart from „classical” sodium pump inhibition have been described. Below we describe some of what appears to be the most pathophysiologically relevant for the development of PE with evidence based on human in vivo and in vitro studies, as well as appropriate animal models.

#### 4.4.1. Vasoconstriction

The most established effect of EDLF is the inhibition of Na^+^/K^+^-ATPase activity. Consequently, this may produce vasoconstrictive effects by increasing intracellular calcium in smooth muscle cells. This mechanism is well-characterized and known as the „Blaustein effect” [27]. α2 isoform of Na^+^/K^+^-ATPase is co-localized with the Na^+^/Ca^2+^-exchanger in smooth muscle cells where these two transport systems appear to function cooperatively [90,91]. Thus, the inhibition of NKA by EDLF induces the elevation of local Na^+^, an increase in local Ca^2+,^ and a change in muscle contractility [92,93]. The physiological function of CS is regulating sodium excretion by inhibiting the sodium pump in the epithelial cells of renal tubules, i.e., adaptive response [67]. Under pathological conditions, for example, in patients with PE, the side effect of primarily adaptive response becomes dominant, and CS causes inhibition of sodium pumps in other tissues, including blood vessels [94].

#### 4.4.2. Vascular Fibrosis

The cornerstone in the pathogenesis of preeclampsia is the abnormal remodeling of uterine spiral arteries and their subsequent fibrosis by various vascular factors, including cardiotonic steroids [95]. Recent research demonstrated that in addition to the vasoconstrictor effects of CS, these compounds directly affect intracellular signaling resulting in vascular fibrosis and impaired vasorelaxation [96]. MBG has been demonstrated to stimulate collagen synthesis and induce fibrosis in cardiac and renal tissues [97] and, in hypertensive humans, correlates with vascular stiffness [98].

Xie et al., first demonstrated that in addition to pumping ions (i.e., “ionic mechanism”), Na^+^/K^+^-ATPase acts as a signal transducer (i.e., “signaling mechanism”). The first major pathway that was identified involves the activation of Src, transactivation of the EGFR, and increased production of reactive oxygen species (ROS) [99].

There is substantial evidence that the profibrotic effect of MBG is attributed to another intracellular pathway, the EGFR-Src-dependent inhibition of Fli-1, a nuclear transcription factor, and a negative collagen-1 regulator [95]. The Na^+^/K^+^-ATPase/Src/EGFR complex activates signaling, which results in the phosphorylation of PKCδ and its translocation to the nucleus, then PKCδ phosphorylates Fli-1, which withdraws the Fli1-induced inhibition of the Col1 promoter and increases procollagen expression and collagen-1 production [100]. The role of Fli-1-dependent fibrosis induced by MBG in preeclampsia is supported by extensive literature.

Based on the observation that MBG induces cardiovascular fibrosis, Nikitina et al., hypothesized that the latter may also be involved in PE. A study of both normal and preeclamptic umbilical arteries explants was conducted. Umbilical arteries collected from women who developed PE exhibited significantly reduced expression of Fli-1, whereas procollagen-1 expression was increased compared to control vessels. Ex vivo treatment of normal umbilical arteries explants with MBG mimicked the effects of preeclampsia, i. e. suppressed Fli-1 and increased collagen-1 expression [67].

These findings were confirmed in later research. In rat aortic explants and vascular smooth muscle cells, pretreatment with MBG decreased the Fli-1 level and resulted in a rise in collagen-1 [96]. In humans, higher plasma MBG level was accompanied by up to a 5-fold decrease in Fli-1 level and up to 4-fold increase in collagen-1 level in the preeclamptic umbilical arteries compared to those from the normal subjects [74,101].

Similar observations were made in placental tissue. Development of PE was associated with a higher placental MBG, a 9-fold decrease in the FLi-1 level, and a 2.5-fold increase in collagen-1 in placentae [89]. Incubation of healthy human umbilical arteries with nanomolar concentrations of MBG resulted in inhibition of Fli1 expression, increased synthesis of collagen-1, and impaired vasorelaxation, mimicking the effects of preeclampsia [67,74,89]. Isolated rings of umbilical arteries collected after delivery in pregnancies complicated by PE exhibited an impaired response to the relaxant effect of sodium nitroprusside in vitro [67,74,101]. This was also observed in aortic rings of NaCl-loaded rat models of PE [96,102]. In a recent study, the silencing of Fli1 in healthy human umbilical arteries was associated with an elevation in procollagen and collagen-1 levels in vascular tissue [103].

While Fli-1 dependent mechanism remains well-established, the significance of another putative mechanism underlying vascular fibrosis in preeclampsia is more conflicting. Ohmaru et al., proposed that TGF-β1 signaling is activated in the fibroblasts in preeclamptic placentas and contributes to fibrosis [104]. Subsequently, a study of NaCl-loaded diabetic rats showed increased TGF-β and decreased Fli-1 in the thoracic aorta, accompanied by elevated MBG plasma levels. The authors suggested that both Fli-1 and TGF-β are implicated in MBG-dependent fibrosis in preeclampsia [102]. However, a recent study did not confirm these observations. While the development of PE was associated with reduced Fli-1 in umbilical arteries and elevated collagen-1, it was accompanied by an absence of changes in TGF-β and collagen-4 levels. Likewise, incubation of umbilical artery rings in the presence of MBG resulted in the reduction of Fli-1 level and concomitant increase in the collagen-1 level, while expression of TGF-β and collagen-4 remained unaffected [74]. These findings suggest that the TGF-β pathway may not be involved in vascular fibrosis in PE, or its significance is minor.

Recent studies demonstrate the clinical importance of fibrosis in the cardiovascular system and placenta [104,105]. As the vasoconstrictive effect via the Blaustein effect is temporary, the Fli-1-dependent fibrosis may be a mechanism implicated in increased long-term cardiovascular risk that persists after delivery in women who have undergone preeclampsia.

#### 4.4.3. Other Mechanisms

Imbalance in vasoactive factors, proangiogenic shift, endothelial damage, inflammation, and apoptosis are some of the other proposed mechanisms implicating the role of EDLF in the pathogenesis of preeclampsia. Since the literature on these mechanisms is less extensive or the evidence is based mainly on studies of animal models of PE, we discussed them collectively.

Altered sensitivity to vasoactive humoral factors contributes to the development of preeclampsia. Prolonged exposure to ouabain enhances the vasoconstrictor mechanisms in the arterial smooth muscle while simultaneously downregulating the endothelial vasorelaxant feedback mechanisms [106]. In one study, intraperitoneal injection of EDLF upregulated endothelin (ET) levels and, consequently, increased the blood pressure of normal female rats. This indicates that changes in the ET system may be one of the mechanisms by which EDLF causes hypertension and organ damage [107]. Increased sensitivity to endogenous α-adrenaline is another mechanism linked to EDLF-induced vasoconstriction [108,109]. Transient stimulation of brain endogenous ouabain (EO) precedes the increase in renal excretion of MBG. In a study on NaCl-loaded Dahl rats, brain EO stimulated peripheral MBG via the angiotensin II receptor type 1 (AT1) pathway and probably via sympathetic activation, causing a hypertensive effect [110]. The latter mechanisms lead to the formation of a nervous–humoral regulatory network involving endothelin (ET), nitric oxide (NO), and the renin–angiotensin–aldosterone system (RAS), among others, which are involved in the development of preeclampsia [111,112].

Another mechanism that may involve cardiotonic steroids in the pathogenesis of PE is their potential ability to alter the angiogenic balance. In patients with PE, levels of sFlt1 and sENG were found to be elevated together with CS [79]. In one study, ouabain and MBG stimulated the release of the antiangiogenic sFlt-1 and decreased the release of the proangiogenic vascular endothelial growth factor (VEGF) and placental growth factor (PlGF) [113]. In contrast, in another study, ouabain down-regulated sFlt1 production by inhibiting hypoxia-inducible factor 1 (HIF-1α) protein expression in the placenta [114].

Preeclampsia is associated with systemic damage to endothelial cells and increased microvascular permeability. Peng et al., proposed that EDLF is the factor causing endothelial damage in preeclampsia. In a recent study comparing 60 women diagnosed with severe PE with healthy pregnant women, EDLF was found to cause vascular endothelial cell damage involving the NF-κB pathway [81].

Finally, EDLF may be implicated in oxidative stress in preeclampsia. The binding of ouabain to the NKA results in the activation of Src, and transactivation of the EGFR, leading to increased production of reactive oxygen species (ROS) [99]. In the ex vivo preeclamptic trophoblast, oxidative stress promotes the transcription of antiangiogenic factors and inhibits the Wnt/β-catenin signaling pathway impairing trophoblast invasiveness [5]. In addition, the MBG induces impairment of cytotrophoblast function via activation of Jnk, p38, and Src leading to increased apoptosis and IL-6 secretion. Regarding the treatment of preeclampsia, these observations could be clinically relevant [115].

### 4.5. EDLFs as a Potential Therapeutic Target in Preeclampsia

#### 4.5.1. In Vitro and Animal Studies

The need for causative treatment of preeclampsia remains unfulfilled, with delivery as the only definitive remedy. While PE is multifactorial and eliminating only one piece of the puzzle likely will not resolve the disease, it may substantially ameliorate its course and prevent complications.

**Figure 1:**
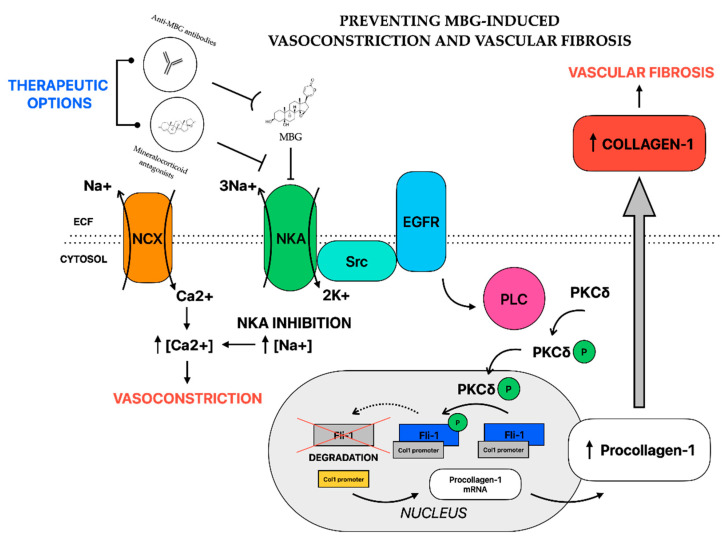
Pathways of MBG-induced vasoconstriction and vascular fibrosis and therapeutic roles of anti-MBG antibodies and mineralocorticoid antagonists via suppressing inhibition of NKA. Abbreviations: EGFR—epidermal growth factor; Fli-1—Friend leukemia virus integration 1; MBG—marinobufagenin; NCX—Na^+^-Ca^2+^-exchanger; NKA—Na^+^/K^+^-ATPase; PKCδ—phosphokinase C δ; PLC—phospholipase C.

Two possible treatment approaches are investigated for neutralizing the negative effects of EDLF in preeclampsia—immunoneutralization and antagonism against receptor [95] (Figure 1). Major of the evidence of the efficacy of this approach is based on in vitro studies and animal models of preeclampsia. As spontaneous preeclampsia is unique to human gestation [5], animal models were established to facilitate investigating biochemical processes underlying the development of preeclampsia. Discussing animal models of PE is beyond the scope of the present paper [5,116,117,118].

The first suggestion that ADA-FAB may reverse the NKA inhibitory effect of EDLF and is a potential therapeutic agent in PE comes from Goodlin, 1987 [119]. Subsequent studies investigated the efficacy of Digibind@ and specific antibodies to MBG and ouabain. Administration of anti-MBG antibodies to NaCl-loaded pregnant Sprague–Dawley rats lowered blood pressure and restored the activity of NKA in thoracic aortae [79]. Likewise, monoclonal antibodies to MBG and ADA-FAB lowered blood pressure and reversed NKA inhibition in vascular tissue in NaCl-loaded pregnant Dahl rats [64]. Studies of specimens collected from humans who developed PE confirmed these findings. Inhibition of NKA in red blood cells (RBC) from patients with preeclampsia is reversed ex vivo by anti-MBG antibodies [47,49,64] and ADA-FAB [49,73]. In contrast, an anti-ouabain antibody was ineffective in restoring NKA activity [47]. ADA-FAB reduced vascular resistance in isolated placental lobules [120] and reversed sodium pump inhibition in preeclamptic placenta [73]. Moreover, anti-MBG antibodies have been demonstrated to correct the angiogenic profile of cytotrophoblast cells induced by MBG [121].

ADA-FAB preparation utilized in many of these studies is no longer commercially available (Digibind@). A new commercial product (DigiFab@) also reverses PE-induced NKA inhibition, has similar cross-activity with both MBG and ouabain and can potentially substitute Digibind@ for immunoneutralization of CTS in patients with PE [49]. The effect of anti-digoxin antibodies in PE is due to cross-reacting with other steroids. In humans, ADA-FAB reversed NKA inhibition induced by multiple cardenolides and bufadienolides. However, digoxin-specific Fab is more than 20-fold more potent in neutralizing ouabain than marinobufagenin [122]. ADA-FAB (Digibind@) was less effective regarding both restoration of NKA activity in human RBCs [47,64] and lowering blood pressure in hypertensive animal models when compared to anit-MBG [64]. Relatively low cross-reactivity with MBG is likely the reason for its limited efficacy. In recent studies, the immunoneutralizing of MBG was demonstrated to prevent its profibrotic effect in preeclampsia. Treatment of diabetic NaCl-loaded rats with anti-MBG mAb-activated NKA prevented increases in aortic weights and returned levels of fibrosis markers to control levels [102]. In NaCl-loaded pregnant Sprague–Dawley rats treated with polyclonal anti-MBG-4 antibodies, a drop in blood pressure was accompanied by a reduction in the decrease in Fli-1 [123]. In umbilical arteries collected from preeclamptic patients, fibrosis, measured with the level of collagen-1, was reversed by preincubation with monoclonal anti-MBG antibody [89].

Another approach to neutralize the EDLF effect is by administration of mineralocorticoid antagonists. Spironolactone, and its active metabolite canrenone, act as competing inhibitors of cardiotonic steroids and sodium pump activity [124]. At therapeutic concentrations, canrenone reduces the ability of EDLF to inhibit the sodium pump [124]. This prevents MBG-induced increase in collagen synthesis, thus, vascular fibrosis [96]. Spironolactone suppresses cardiac fibrosis induced in rat models by chronic administration of MBG [125]. In rat aortic explants, canrenone, an active metabolite of spironolactone, blocked the effect of MBG on collagen synthesis and restored sensitivity to sodium nitroprusside [96]. Resistant hypertension in humans was accompanied by elevated plasma MBG and reduced NKA activity. In those patients, six-month administration of spironolactone was associated with decreased pulse-wave velocity and blood pressure with restoration of NKA activity. The MBG levels remained unchanged [96]. In a recent study, human umbilical arteries obtained from patients with PE were treated in vitro with canrenone, which blocked the effect on Fli-1 and collagen-1 levels. These results were reproduced for normal umbilical arteries pretreated with MBG [74]. As spironolactone administration is associated with the feminization of male fetuses, its administration during pregnancy may not be suitable [126]. In animal studies, eplerenone, another mineralocorticoid antagonist, has not been associated with adverse reactions during pregnancy [95]. While data on its use in human pregnancy are lacking, future studies may be warranted.

#### 4.5.2. Case Reports and Clinical Trails

A series of preclinical studies that explored the role of EDLF in pathogenesis and ADA-FAB therapeutic potential in preeclampsia are discussed above. Clinical evidence for the therapeutic potential of digoxin-immune Fab (DIF) in preeclampsia comes from three single case reports and two completed clinical trials. The most recent trial in this indication was prematurely terminated due to futility, and no further trials have been initiated.

The first anecdotal report of ADA-FAB therapeutic potential in preeclampsia came from Goodlin, 1988. Primipara, in 26 weeks, presented with severe preeclampsia and was administered two doses of Digibind@, which resulted in a transient hypotensive response [50]. Adair et al., in 1996, reported a single case of second-trimester twin pregnancy complicated with preeclampsia. An elevated serum level of digoxin-like immune factor was found, and continuous intravenous infusion of digoxin-binding immunoglobulin lowered blood pressure [127]. Subsequently, in 2009, Adair et al., reported a case of primipara in 30 weeks, who presented with eclampsia. ADA-FAB was administered in a repeated fixed schedule every 6 h. Dosing was based on the treatment regimen reported in two prior subjects. Following the study, drug infusions reduction in mean arterial pressure (MAP) was noted. Furthermore, there was an improvement in neurological exam, renal function, and umbilical artery Doppler waveforms [128].

These encouraging yet anecdotal findings led to the first double-blind, placebo-controlled, randomized clinical trial investigating the potential blood pressure effects of ADA-FAB (Digibind@) treatment in postpartum subjects [129]. As blood pressure does not normalize immediately after delivery [130], the study drug was administered during the immediate postpartum period to avoid fetal exposure to unknown effects of digoxin-immune Fab (DIF). A total of 26 subjects who met ACOG diagnostic criteria of severe preeclampsia [131] were enrolled and randomized. After delivery, single doses of DIF (Digibind@) or placebo were administered within 15 minutes of clamping the umbilical cord. The primary outcome, an effect on the mean arterial pressure (MAP) during the first 6 h postpartum, was negative. However, a post hoc analysis noted significantly lower MAP over the first 4 h of treatment. Moreover, no subject from the treatment group required conventional antihypertensive therapy during 24 h following the study drug administration, compared with 46% of placebo subjects. Furthermore, there was a tendency for higher creatinine clearance in the DIF group, although this change was of borderline statistical significance. No treatment-related adverse events were noted [129].

DEEP (DIF Efficacy Evaluation in Preeclampsia) Study was a subsequent Phase 2b study performed in a group of antepartum subjects with severe preeclampsia [132]. In total, 51 subjects between 24 and 34 weeks of gestational age diagnosed with severe preeclampsia as defined by ACOG were enrolled. The study drug was given intravenously every 6 h for eight doses during the 48-h treatment phase. Primary endpoints were the use of antihypertensives and creatinine clearance. Although the use of antihypertensives was similar between groups (46 and 52%, for DIF and placebo, respectively), there was a significant advantage over placebo regarding creatinine clearance in the DIF-treated group. Additionally, serum sodium pump inhibition was decreased with DIF compared with placebo at 24 h after treatment initiation. The authors proposed that these results suggest that DIF prevents a decline in renal function in severe preeclampsia by neutralizing EDLF [132]. A secondary analysis of data derived from the DEEP study was performed [133]. The objective was to determine if there is an improvement in maternal and neonatal outcomes in women serum positive for EDLF treated with DIF. Indeed, in the EDLF-positive women treated with DIF, there was a lower rate of pulmonary edema, and the infants had lower rates of intraventricular hemorrhage (IVH). DIF treatment effectively restored the sodium pump activity in this group. The authors concluded that women with severe preeclampsia who were EDLF-positive may benefit from the use of DIF, and the treatment can improve maternal and neonatal outcomes [133].

In 2017, Phase 2b/3a Study of the Efficacy and Safety of AMAG-423 (Digoxin Immune Fab) in Antepartum Subjects with Severe Preeclampsia was started (NCT03008616). Subjects diagnosed with severe preeclampsia between 23 and 32 weeks for whom expectant management was an option were enrolled, allocated to two groups, and received either DIF or placebo every 6 h for 4 days, a total of 16 doses. The primary endpoint was a proportion of infants with IVH, NEC, or death by 36 weeks corrected gestational age. After recruiting 59 patients, the study was terminated early for futility. There were no safety concerns raised. Notably, DIF appears to be well tolerated and safe for both mother and fetus. In any of conducted studies, there were no treatment-related adverse events.

## 5. Discussion

Preeclampsia, the most severe hypertensive disorder of pregnancy, is the leading cause of maternal death, severe maternal morbidity, and prematurity. Despite advances in managing preeclampsia, the need for causative treatment remains unfulfilled. A closer look at the pathogenesis of preeclampsia may bring new treatment options. As it is generally accepted that preeclampsia processes according to a two-stage model, the pathogenesis of this complex disorder remains elusive. Endogenous digitalis-like factors (EDLF) are the Na^+^/K^+^-ATPase (NKA) inhibitors implicated in the pathogenesis of preeclampsia and are one but essential piece of this puzzle. The most attention on cardiotonic steroids’ role in PE focused on bufadienolide—marinobufagenin (MBG) and cardenolide—endogenous ouabain (EO).

The Na^+^/K^+^-ATPase (NKA), the ubiquitous active transport system of sodium and potassium, maintains a transmembrane ionic gradient and has been identified as a scaffolding and signaling protein. Endogenous digitalis-like factors (EDLF) are the ligand for the NKA as the receptor and are involved in the pathogenesis of preeclampsia through interaction with NKA. EDLF exhibits differential affinity for specific α-subunits of NKA, hence acting in a tissue-specific fashion. Thus, α1 NKA, predominant in vascular smooth muscle, is inhibited by physiologically relevant concentrations of MBG, while α2 NKA, an isoform localized in the brain, is more sensitive to EO. Binding cardiotonic steroids to its receptor site on NKA results in a classic “ionic mechanism” and contributes to vasoconstriction, hence blood pressure elevation, via interaction with a^+^/Ca^2+^-exchanger (Blaustein effect). Recent discoveries demonstrated that binding EDLF to NKA, in addition to vasoconstriction, may produce more complex effects via a “signaling mechanism”. Preeclampsia is a disorder associated with hypertension, vascular fibrosis, systemic inflammation, and endothelial damage, and these mechanisms may be attributed to the dual role of NKA. The most established signaling mechanism involved in PE is the EGFR-Src-dependent inhibition of Fli-1, resulting in vascular fibrosis. MBG, via interaction with NKA, inhibits Fli-1, a negative regulator of collagen-1 synthesis, and stimulates collagen production. Furthermore, TGF-β was demonstrated to contribute to MBG-dependent fibrosis in preeclampsia, but the results are conflicting. Other identified pathways are endothelial damage involving the NF-κB signaling, impairment of cytotrophoblast function via activation of Jnk, p38, and Src leading to increased apoptosis and IL-6 secretion, activation of the renin–angiotensin–aldosterone system (RAS), promoted vasoconstriction with simultaneously downregulated vasodilatation, and alterations in angiogenic balance. Studies have confirmed elevated digitalis-like immunoreactivity and Na^+^/K^+^-ATPase inhibitory activity in preeclampsia. Elevated serum levels of EDLF correlate with the severity of preeclampsia, and the placenta is the site of synthesis of cardiotonic steroids during pregnancy. MBG is markedly elevated in the serum and placenta of preeclamptic women and likely is the primary cardiotonic steroid implicated in the pathogenesis of preeclampsia. Ouabain’s role in the development of PE is more elusive. NKA isoforms in central nervous systems are more sensitive to ouabain, and there is evidence for the neuroendocrine source of endogenous ouabain, which may have a rather central than peripheral role. It is supported by a study that demonstrated that transient stimulation of brain EO precedes the increase in renal excretion of MBG.

Two potential treatment approaches in antagonizing the effects of EDLF in PE are investigated—immunoneutralization and antagonism against receptors. In vitro studies and animal models have shown that anti-MBG antibodies and ADA-FAB can reverse the NKA inhibitory effect of EDLF in preeclampsia, and anti-MBG antibodies are also effective in reversing vascular fibrosis. Clinical studies utilized Digibind as an immunoneutalizing agent towards EDLF. Concerning the antihypertensive effect, the results of these studies are conflicting, which may be, at least to some extent, due to the relatively low affinity of Digibind to MBG. Moreover, a secondary analysis of the DEEP study showed that DIF may be beneficial when administered to EDLF-positive women. Identifying this group may be an interesting novel approach to managing preeclampsia, and future studies are warranted. Mineralocorticoid antagonists, such as spironolactone and canrenone, can also neutralize the EDLF effect by reducing the sodium pump activity and preventing MBG-induced collagen synthesis. Women who have undergone preeclampsia have increased cardiovascular risk later in life. Since vascular fibrosis may be responsible for long-term consequences, neutralizing MBG during pregnancy could mitigate this impact. Further research is needed to determine the effectiveness and safety of these approaches in human pregnancy.

## Figures and Tables

**Table 1:** Cross-immunoreactivity of Digibind, DigiFab, anti-ouabain and anti-MBG antibodies (%) (according to Ishkaraeva-Yakovleva et al.).

Cross-Reactant	Digibind (ADA-FAB)	DigiFab (ADA-FAB)	Anti-O Polyclonal Antibody	3E9 Anti-MBG Monoclonal Antibody	4G4 Anti-MBG Monoclonal Antibody
Digoxin	100	100	1.8	1.8	0.03
Digitoxin	1.4	1.3	0.47	0.7	<0.001
Ouabain	0.4	0.14	100	0.02	0.005
MBG	0.2	0.29	0.036	100	100
Marinobufotoxin	0.06	0.02	0.06	4	43
Telocinobufagin	1.0	0.34	0.02	7	14
Bufalin	2.7	0.9	0.10	0.3	0.08
Cinobufagin	0.02	0.03	0.02	1.4	0.07
Resibufagenin	1	0.02	0.15	0.5	0.5
Proscillaridin-A	1.46	0.2	0.03	3	<0.001
Prednisone	<0.001	<0.001	0.001	<0.001	<0.001
Progesterone	<0.001	<0.001	0.002	<0.01	<0.001

## Data Availability

The data presented in this study are available on request from the corresponding author.
